# Supplementary figures and images for: Non-invasive evaluation of the equine gastrointestinal mucosal transcriptome
Source: PLoS One. 2020 Mar 16;15(3):e0229797. doi: 10.1371/journal.pone.0229797 (PMC7075554; doi:10.1371/journal.pone.0229797)

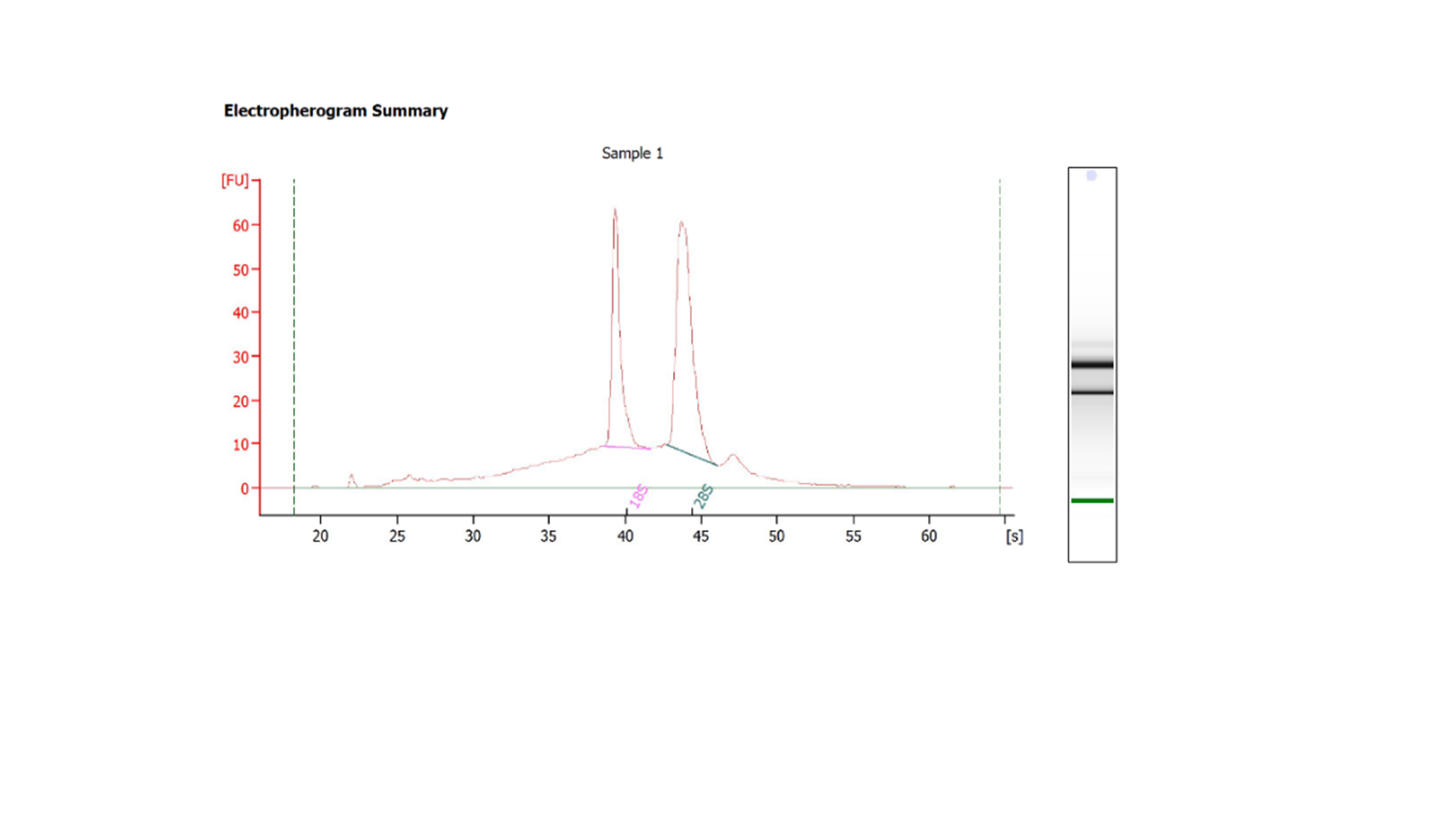

Supplement: S1 Fig — (TIF) [file pone.0229797.s001.tif]

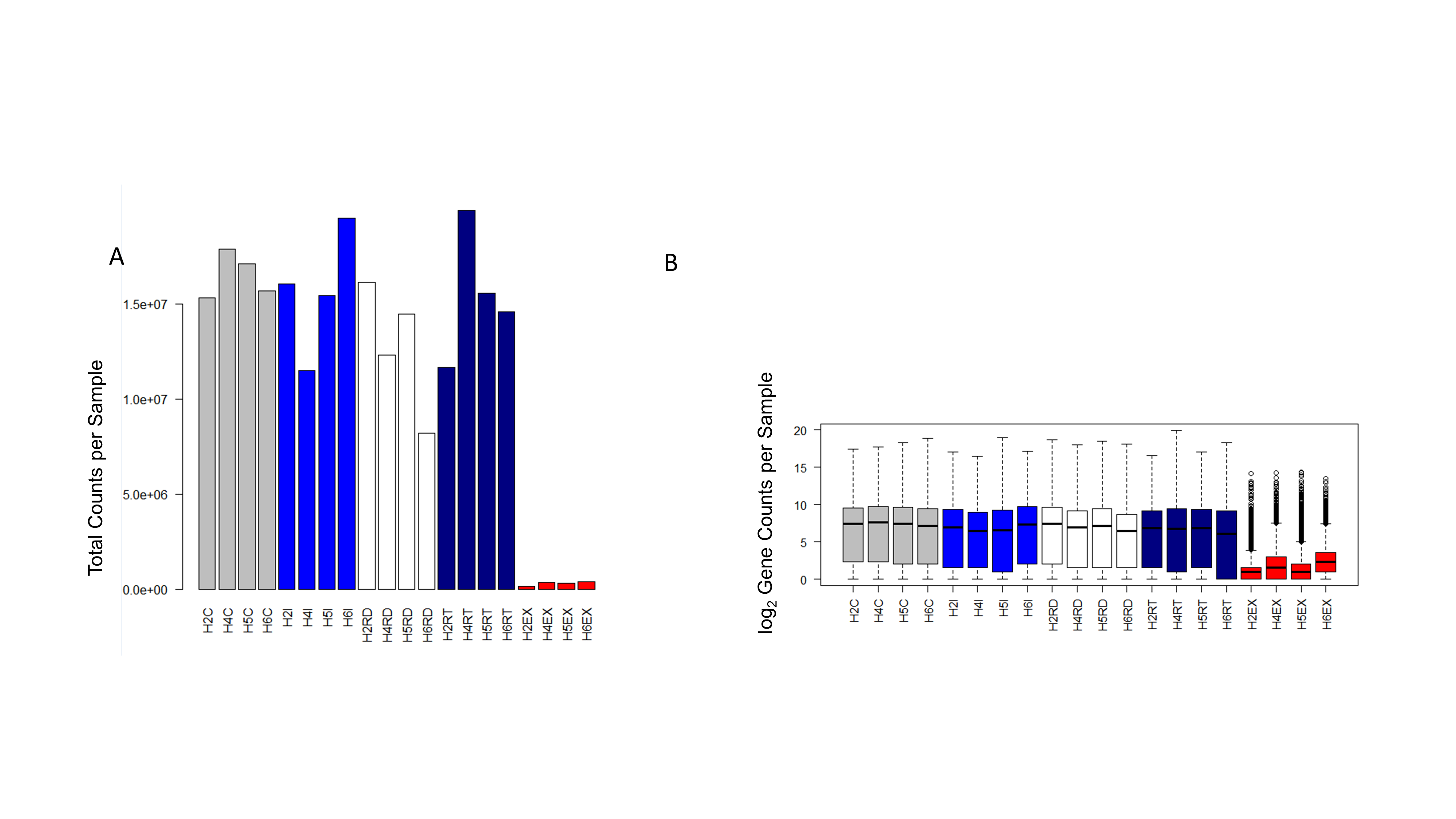

Supplement: S2 Fig — A) Bar plots of total counts per sample from both tissue and feces. B) Boxplots of log2 of gene counts per sample from both tissue and feces. Samples are identified by sample number (i.e., H2, H4, H5, H6) and sample source (i.e. EX = exfoliome, I = ileum, RD = right dorsal colon, C = cecum, RT = rectum). (TIF) [file pone.0229797.s002.tif]
