# Supplementary material for: Non-invasive evaluation of the equine gastrointestinal mucosal transcriptome
Source: PLoS One. 2020 Mar 16;15(3):e0229797. doi: 10.1371/journal.pone.0229797 (PMC7075554; doi:10.1371/journal.pone.0229797)
Supplement: Attachment — Submitted filename: Response to Reviewers_2-12-20.docx [file pone.0229797.s004.docx]

February 12^th^, 2020

Editorial Board of PlosOne

Subject:	Manuscript entitled, “Non-invasive evaluation of the equine gastrointestinal mucosal transcriptome” (PONE-D-19-27855R1)

Dear Members of the Editorial and Review Board:

Thank you for your careful, thorough and thoughtful review of the manuscript. We have carefully considered and responded to each of the recommendations. Each comment posed by the reviewers had been addressed below with corresponding changes and improvements to the manuscript.

**Reviewer #1**:

**No response**

**Reviewer #2**:

**Overall, this is a significantly improved manuscript. The authors have addressed almost all of this reviewer's concerns.**

**A few points that need to be addressed.**

**Abstract:**

**Line 41: There was a coorelation, questionable if this could be stated as 'strong', please revise.**

*Authors’ Response:* This was revised as requested. We apologize for missing this over statement in the initial revision.

**Results:**

**Line 223: This statement is what is mis-represented in the abstract. As written, “There was a strong and significant coorelation of all tissues…an, although of lesser magnitude, significant correlation between the exfoliome and each tissue source”.**

*Authors’ Response:* This was revised in the abstract as requested.

**Reviewer #3**:

**No response**

We again thank you for the time and effort dedicated to this review process. We hope you agree that the clarity and quality of the manuscript has substantially improved. For any questions or concerns regarding this submission, please contact me by telephone at my office (979-845-3541) or by email ([cwhitfield@cvm.tamu.edu](mailto:cwhitfield@cvm.tamu.edu)). Thank you for your consideration.

Sincerely,

Canaan Whitfield-Cargile, Assistant Professor

Department of Large Animal Clinical Sciences

College of Veterinary Medicine & Biomedical Sciences

Texas A&M University

College Station, TX 77843-4475
